# Governing the allocation of scarce resources: is health care no longer a special case?

**DOI:** 10.1186/2045-4015-3-23

**Published:** 2014-06-24

**Authors:** David Chinitz

**Affiliations:** 1 School of Public Health, Hebrew University, Hebrew, Hadassah

## Abstract

Health care rationing has always been seen as a wicked problem, somehow special and not subject to the same rules of engagement as other policy areas. The article by Philip Sax makes a strong contribution by placing one prominent part of rationing, determination of a national drug formulary, into the larger political economy context of Israeli policy making. While one can argue with some of Sax’s implied conclusions, his analysis provides a great platform not only to understand governance of this difficult area, but also to better govern in the future.

## 

Health care rationing is one of the least comfortable topics to address, especially for politicians charged with making health resource allocation decisions, but also for health policy analysts. Nobody wants to “play God.” This reticence was captured in the breakthrough book, *The Painful Prescription* by Aaron and Schwartz from 1986 [1]. That volume described how rationing of health care in both the US and the UK was done implicitly, in the US by lack of access to health insurance, and in the UK by subtle denial of services such as renal dialysis to patients over 65. That book, as well as *Tragic Choices* by Guido Calabresi [2], were among those that opened a more explicit discussion about rationing in health systems.

Beginning in the mid 1990s, the literature on rationing grew and the International Society for Priority Setting in Health Care began to meet every two years to discuss the issue [3]. The main lines of discussion turned on substance and process. Regarding the first, economists and ethicists (though not often in concert) led the way, seeking rules for rationing. Some of the credos that emerged included:

• Benefit cost ratios should be maximized

• Benefits can be defined in terms of quality adjusted life years

• Public preferences for health services can be measured and used to inform rationing

• Health resources should be allocated so as to equalize opportunity across members of society

On the process side, the leading figure was perhaps Rudolph Klein [4], who appealed repeatedly to economists and technocrats to focus on the design of legitimate, accountable, public institutions charged with making rationing decisions. Norman Daniels [5] developed what is probably the most stable paradigm for health care priority setting: Accountability for Reasonableness, which emphasized that decision making processes should be transparent and reasonable, regardless of the substantive outcomes they produce.

Because of the sensitive nature of health care rationing, these twin strands of analysis stood apart from larger political contexts. Comparisons between countries regarding rationing often turned on matters of culture. Analysts would occasionally quip that policy mechanisms routinely deal with life and death decisions, such as in investment in traffic safety, but the mechanisms of governance that seemed to apply to most areas of social policy, even those with health implications, somehow were left tangential to discussions of health care rationing.

However, as a number of countries have taken different paths to making health care priority setting more explicit, the linkages to the overall machinery of policy making have become more apparent. In other words, health care rationing can now be addressed as a “regular” policy issue, subject to the same theories regarding interest groups, public choice, regulatory behavior and political leadership that are considered salient in any policy area.

The paper by Sax [6] on which this commentary is based, provides an excellent example. Almost without batting an eyelash, the author seeks to explain health care rationing, in particular regarding pharmaceuticals, not as a “sacred” special ethical issue, but as a matter of governance. Using the Israeli case, Sax nicely demonstrates how the politics of access to drugs may be morally and ethically charged (see under “death panels”) but remain politics nonetheless.

Sax uses political theory in an effective way to trace the evolution of governance of pharmaceutical policy. He identifies correctly, in the view of this author, three stages of development. The first stage consists of the inchoate market for drugs that existed prior to enactment of National Health Insurance (NHI) in Israel in 1995. During this first period, policy and decision making at various levels in the health system regarding pharmaceuticals emphasized safety and pricing. The situation in Israel was one of large purchasers, the four major health maintenance organizations (HMO) and the government, who purchased and allocated drugs in bulk. Most of the action in the pharmaceutical sector was around verifying the safety of drugs, based on approval in other countries by agencies such as the Food and Drug Administration (FDA) in the US, and on pricing. This parallels the situation in other countries, such as the US, the Netherlands and Germany, in which health technology assessment (HTA) was only beginning to be deployed regarding medical interventions, and mechanisms such as reference pricing were just beginning to be mandated by publicly financed health insurance programs [7].

The second stage, ensuing with the implementation of NHI, moved the system from a market with large players, to a more hierarchical situation in which government established a national formulary that obligated the four HMOs. Born out of the fundamental pillars of the law, the formulary – arguably the most visible part of the standard basket of health services mandated by the law – combined the desire for financial viability and accountability with legal responsibility to provide a clear set of benefits. Whereas before NHI, HMOs had wide latitude in determining what drugs would be available, after the passage of NHI access to pharmaceuticals was determined on a national basis. As Sax points out, partly due to public pressure, a multi-sector Committee to Update the Basket of Health Services was created in 1998 and has become the institutional manifestation of the hierarchical governance introduced in the pharmaceutical sector. Sax compares the Israeli situation to the role of the Pharmaceutical Management Committee (PHARMAC) in New Zealand, hinting at preference for the latter because of its harder bargain driving vis a vis drug companies. Be that as it may, Israel is a social insurance based system, while New Zealand is a Beveridge system and therefore less pluralistic in its overall governance of health care. Given that, the degree of hierarchy characterizing governance of the Israeli pharmaceutical sector is impressive.

Sax’s third stage brings to bear network theory, which describes and explains the web of stakeholder relationships that inevitably emerge within hierarchical governance. Pharmaceutical companies, regulators, HMOs, and physicians interact within the official process of updating the basket of services, each trying to advance their own agenda. Sax labels this a kind of “neo corporatist” arrangement, in which interest groups interact under government auspices to determine policy outcomes. The implication in the article is that the web of interacting stakeholders is associated with lack of participation of the broader public in the priority setting process, and lack of transparency as to how decisions are being made. In particular, Sax concerns himself with the degree to which allocation decisions made by government, represented by the Committee to Update the Basic Basket, are implemented by the HMOs.

There are several problems with this last part of the analysis. First of all, corporatism implies that the stakeholders at the table and in the game represent a broad array of the key interested parties, including the general public. It should be remembered that citizens’ rights groups, as well as disease oriented interest groups exert leverage on the Committee, meaning that there is public input. Second, Sax seems to imply that pharmaceutical companies have an inside track in the decision making process. However, it should be remembered that on average, only about 70 of 600 candidates to be included in the basket of health services are ultimately inducted. This suggests that even if pharmaceutical companies are equipped to exert undue influence, there are winners and losers among the pharmaceutical companies themselves, in effect not only cancelling out the strength of these special interests, but making them dependent on the members of the Committee and the degree of public support for their favored candidates for coverage. Finally, while it is true that the strategy of HMOs in influencing the decisions of the Committee and how they allocate additional funds are not transparent, the funds are used for health services one way or the other which means that “rents” in the form of over budgeting of specific drugs are not a dead weight welfare loss to the health system.. From a transaction cost point of view, the costs and benefits of increased transparency need to be weighed. One possible conclusion is that the process of admitting new drugs into the basket serves to make tough decisions in allocating the increment of funds made available each year by the government, but beyond this it is efficient to leave the actual allocations to the HMOs.

Sax argues that the process has emphasized medical services, to the exclusion of health promotion, prevention and population health targeted services. It could be argued, however, that the increasing “education” of the public to the need for priority setting, even in health care, which Sax himself references, provides a basis on which to alter allocations in the direction of population health in the future. Indeed, certain treatments, such as behavioral smoking cessation programs, have been given priority by the Committee and included in the basket.

The invaluable contribution of this paper is placing health care priority setting in a broader political theoretic framework. The transition that took place in Israel is well captured by the market to hierarchy to network evolution put forward in the paper. The paper creates a platform for identifying the current challenges facing Israeli pharmaceutical governance, including the degree to which more transparency is needed and the problem of “medicalization” at the expense of public health. The governance musculature developed in Israel in the tough area of health resource allocation provides an interesting paradigm for other health systems, just as it can be the basis for further positive incremental change.

Commentary on The shaping of pharmaceutical governance: the Israeli case

By Philip Sax 2014, **3**:16

## Competing interests

The author declares that he has no competing interests.

## Author information

David Chinitz is Professor of Health Policy and Management at the School of Public Health, Hebrew University-Hadassah and Immediate Past President of the International Society for Priority Setting in Health Care.
